# Site-Directed Mutagenesis Study Revealed Three Important Residues in Hc-DAF-22, a Key Enzyme Regulating Diapause of *Haemonchus contortus*

**DOI:** 10.3389/fmicb.2017.02176

**Published:** 2017-11-08

**Authors:** Yan Huang, Xiuping Zheng, Hongli Zhang, Haojie Ding, Xiaolu Guo, Yi Yang, Xueqiu Chen, Qianjin Zhou, Aifang Du

**Affiliations:** ^1^College of Animal Sciences, Zhejiang Provincial Key Laboratory of Preventive Veterinary Medicine, Zhejiang University, Hangzhou, China; ^2^Zhejiang Center of Animal Disease Control, Hangzhou, China; ^3^Faculty of Life Science and Biotechnology, Ningbo University, Ningbo, China

**Keywords:** *Haemonchus contortus*, diapause, *daf-22*, site-directed mutagenesis, *Caenorhabditis elegans*

## Abstract

*Haemonchus contortus* (*H. contortus*) is one of the most important parasites of small ruminants, especially goats and sheep. The complex life cycle of this nematode is a main obstacle for the control and prevention of haemonchosis. So far, a special form of arrested development called diapause different from the dauer stage in *Caenorhabditis elegans* (*C. elegans*) has been found in many parasitic nematodes. In our previous study, we have characterized a novel gene *Hc-daf-22* from *H. contortus* sharing high homology with *Ce-daf-22* and functional analysis showed this gene has similar biological function with *Ce-daf-22*. In this study, *Hc-daf-22* mutants were constructed using site-directed mutagenesis, and carried out rescue experiments, RNA interference (RNAi) experiments and *in vitro* enzyme activity analysis with the mutants to further explore the precise function site of Hc-DAF-22. The results showed that *Hc-daf-22* mutants could be expressed in the rescued *ok693* worms and the expression positions were mainly in the intestine which was identical with that of *Hc-daf-22* rescued worms. Through lipid staining we found that *Hc-daf-22* could rescue *daf-22* mutant (*ok693*) from the fatty acid metabolism deficiency while *Hc-daf-22* mutants failed. Brood size and body length analyses in rescue experiment along with body length and life span analyses in RNAi experiment elucidated that *Hc-daf-22* resembled *Ce-daf-22* in effecting the development and capacity of *C. elegans* and mutants impaired the function of *Hc-daf-22*. Together with the protease activity assay, this research revealed three important active resides 84C/299H/349H in Hc-DAF-22 by site-directed mutagenesis.

## Introduction

*Haemonchus contortus* is the major gastrointestinal nematode settled in the abomasum mucosa of small ruminants and caused serious anemia and diarrhea to the host by feeding on blood and mucosa (Veglia, 1915; Besier et al., 2016a). The control and prevention of haemonchosis in goat and sheep need organic combination of many integrated parasite management (IPM) approaches including anthelmintic treatment, grazing management and nutritional management (Kelly et al., 2010; Terrill et al., 2012; Besier et al., 2016b). Meanwhile genetic selection against *H. contortus*, biological control (Sanyal, 2001; Da Silva et al., 2017), alternative anthelmintic compounds and vaccine are novel attempts and some were confirmed to be efficacious (Elandalousi et al., 2013; Bassetto and Amarante, 2015; Morais-Costa et al., 2016). Although three main anthelmintic drug classes (benzimidazoles, imidazothiazoles and macrocyclic lactones) and a recently introduced aminoacetonitrile derivative class used to control haemonchosis are still effective for many field populations (Kearney et al., 2016), more and more anthelmintic resistant strains have been reported worldwide (Kotze and Prichard, 2016; Kumar and Singh, 2016; Leite Dos Santos et al., 2017). Frequent human-mediated invasion of *H. contortus* between different cities and countries and the special mating feature (one female can mate with 4–5 males) together would largely facilitate the spread of anthelmintic resistance, making it a great threaten to both animal husbandry and human health. Therefore, exploring new candidate vaccine molecules and targets of alternative drug are important in this field.

The free living nematode *C. elegans* has been widely used as a model organism in various fields such as cell biology research (Jenzer et al., 2015; Maglioni and Ventura, 2016), drugs and chemistry compounds screen (Racz et al., 2017; Ziehm et al., 2017) and functional analyses of parasitic nematodes (Guo et al., 2016; Yan et al., 2016). This organism has a very short life cycle of about 3 days per generation (egg-to-adult) and transparent appearance, making it easy to maintain and observe in laboratory conditions. Phylogenetic analysis showed that *H. contortus* had very close relationship with *C. elegans*, as they all belong to the Clade V (Blaxter et al., 1998), indicating that these two nematodes might have some similarities in gene functions, thus making *C. elegans* a powerful tool for *H. contortus* research.

The model organism *C. elegans* possess both mitochondrial and peroxisomal beta-oxidation pathways and the later one is the major way to synthesize dauer pheromone or daumone which are based on ascarylose with fatty-acid-like side chains of different lengths (von Reuss et al., 2012). Daumone is a kind of chemical signal directing nematodes to enter the dauer state under unfavorable conditions (Wei et al., 2016). In *C. elegans* during the synthesis of dauer pheromone, long-chain fatty acids and very long-chain fatty acids are catalyzed successively by the acyl-CoA oxidase 1 (ACOX-1), the enoyl CoA-hydratase MAOC-1, Ce-DHS-28 and Ce-DAF-22 finally into ascarosides (Gallegos et al., 2001). In *Ce-daf-22* mutant (*ok693*), daumone biosynthesis is defective and thus causes dauer deficiency and the massive accumulation of fatty acids in the worms reduces worm life spans (Joo et al., 2009).

*Haemonchus contortus* could enter another form of arrested development stage, diapause, responding to many reasons including seasonal event such as changes in temperature and photoperiod (Sommerville and Davey, 2002; Rose et al., 2016), low oxygen tension and host immune response (Blitz and Gibbs, 1972a). This behavior could help parasites adapt to different environments. In theory, blocking this pathway can largely improve the control and prevention of haemonchosis and reduce the spring rise phenomenon (Blitz and Gibbs, 1972b), but complete knowledge of parasite diapause is still lacking and needs to be further investigated. Mass spectrometry screen for ascarosides carried out in both free-living and parasitic nematodes revealed that nematodes have a conserved family of signaling molecules and different nematode species produces distinct but overlapping sets of ascarosides indicating that parasites may bear some resemblance with *C. elegans* in the daumone biosynthesis pathways (Choe et al., 2012). Markov and colleagues found that the thiolase *daf-22* involved in *C. elegans* peroxisomal beta-oxidation has two isoforms in *Pristionchus pacificus* (*Ppa-daf-22.1* and *Ppa-daf-22.2*), and they revealed the complexity of functional conservation and divergence with *Ce-daf-22* using the CRISPR/Cas9 system (Markov et al., 2016).

In our previous study, we have characterized a novel gene *Hc-daf-22* from *H. contortus* sharing high homology with *Ce-daf-22* and functional analysis showed this gene has similar biological function with *Ce-daf-22* (Guo et al., 2016). To further explore the precise function site of Hc-DAF-22, we constructed *Hc-daf-22* mutants using site-directed mutagenesis, and carried out rescue experiments, RNAi experiments and enzyme activity analysis with the mutants to see whether certain site mutation influenced the function of Hc-DAF-22.

## Materials and Methods

### Nematode Strains and Maintenance

N_2_-Bristol and *Ce-daf-22* mutant strain (*ok693*) were acquired from the Caenorhabditis Genetics Center (CGC), maintained on NGM petri plates feeding on *Escherichia coli* OP50 at 20°C following standard protocols (Stiernagle, 2006). *H. contortus* (ZJ strain) were isolated and stored in liquid nitrogen previously.

### Construction of *Hc-daf-22* Mutants Using Site-Directed Mutagenesis

Total RNA was extracted from frozen samples of *H. contortus*. Full-length cDNA sequence of *Hc-daf-22* was amplified by RT-PCR using primers designed according to the available gene sequence on NCBI (GenBank ID: HQ738470.1), and then cloned into the pET-22b vector. Possible functional sites were predicted by Prosite (Bairoch, 1993)^1^ and 84C, 299H and 349H were chosen as the target residues. Mutations for each target residues were introduced using the QuikChange Site-directed Mutagenesis kit with designed primer pairs (**Table 1**) and pET-22b-*Hc-daf-22* as a template (Wang and Malcolm, 2002). The PCR products were digested with DpnI for 2 h at 37°C, and were then transformed into *E. coli* TOP10. After identified by PCR, the mutants were further verified through nucleotide sequencing (Biosune, Shanghai, China). For the rescue and RNAi experiments, *Hc-daf-22* mutants (*Hc-daf-22*-84S, *Hc-daf*-22-299A and *Hc-daf-22*-349A) were amplified from the expression vectors and cloned into pPD95_77 and L4440 respectively.

**Table 1:** Primers used in this study.

Primer ID	Primer sequence 5′–3′
*Hc-daf-22*-F	CGCGAATTCTAAATGGGTAAATCAAAAGTATATGT
*Hc-daf-22*-R	GCCGTCGACGATTTTCGCTTTGAGCATTT
*Hc-daf-22*-84S-F	TGAACAATCCCTGCGCTTCTGG
*Hc-daf-22*-84S-R	AGAAGCGCAGGGATTGTTCACGT
*Hc-daf-22*-299A-F	TTATCGAACTTGCTGACTGTTTTGCACC
*Hc-daf-22*-299A-R	TGCAAAACAGTCAGCAAGTTCGATAACC
*Hc-daf-22*-349A-F	AATTTCTAAAGGAGCCCCCATCGG
*Hc-daf-22*-349A-R	TCCTGTCGCACCGATGGGGGCTCCTTTAGAAATTGATCC
*Ce-daf-22*QF	GTTGGAGTCGGTATGACA AAG
*Ce-daf-22*QR	CGGTAAGTCCAACCTCATATAG
*Ce-actin-1*F	GGAATGTGCAAGGCCGGAT
*Ce-actin-1*R	ACCTCCTGGATTGGGCCTC

*The underlines nucleotides showed the positions where the mutations were introduced*.

### Rescue of *Ce-daf-22* Mutant *ok693* with *Hc-daf-22* and Three Mutants

To analyze the rescue efficiency of *Hc-daf-22* and its three mutants for the *Ce-daf-22* mutant *ok693*, four rescue plasmids were constructed with the promoter of *Ce-daf-22* upstream and the GFP downstream. The *ok693* L4s were picked from NGM plates and transferred into another clean NGM plate 12–14 h before microinjection. Rescue plasmids, together with the marker plasmid pRF4 (containing a dominant mutant allele of *rol-6* gene, presenting a left rolling phenotype) were injected into the gonad of young, adult *ok693 C. elegans* hermaphrodites as described (Mello et al., 1991) separately. The final concentration of each plasmid was 50 ng/ml. The F2 and subsequent offspring with a rolling phenotype were chosen for the following fluorescent microscope examination, Oli-Red-O staining and developmental analysis. The measurement of body length and brood size were carried out following the protocol according to Morck and Wong (Wong et al., 1995; Morck and Pilon, 2006). Ten worms were picked randomly for the measurement and the rescue experiment was performed in triplicate.

### Lipid Staining

Oil-red-O staining was performed as previously described (O’Rourke et al., 2009). Briefly, 200–300 adult worms were collected from the NGM plates and transfered into 1 ml centrifuge tubes, washed with PBS (pH 7.4) three times. The supernatant was discarded once worms settled down by gravity. For permeabilization of the worm cuticle, animals were incubated in the mixture of 120 μl PBS and 120 μl 2× the Modified Ruvkun’s witches brew (MRWB) buffer containing 2% paraformaldehyde (PFA) for 1 h at room temperature with gentle rocking. To remove PFA, worms were allowed to settle down by gravity, supernatant was aspirated, and worms were washed with PBS. Then 60% isopropanol was used to resuspend and dehydrate the worms by incubating for 15 min at room temperature. Animals were stained in 1 ml 60% Oli-Red-O dyeing solution overnight with gentle rocking after removal of isopropanol. Dye was discarded when worms had settled by gravity, and 200 μl PBS-0.01% Triton X-100 was added to resuspend worms. Afterward worms were mounted on slides and viewed with a Nikon camera outfitted with differential interference contrast (DIC) optics.

### RNAi Feeding Experiments

RNAi feeding experiments were carried out as described by Lendner et al. (2008). *Hc-daf-22* full-length sequence and three mutant sequences (*Hc-daf-22*-84S, *Hc-daf-22*-299A and *Hc-daf-22*-349A) were cloned into the RNAi feeding plasmid L4440 and transformed into HT115 (DE3) respectively. The RNAi petri plates were made of NGM (Nematode Growth Medium) containing 100 μg/ml ampicillin and 1 mM Isopropyl β-D-thiogalactoside (IPTG) and dried at room temperature for 5 days before use. Fresh RNAi strains were cultured in LB containing 100 μg/ml ampicillin overnight and then spread onto RNAi petri plates, dried at room temperature overnight and stored at 4°C. Synchronized L1 *C. elegans* were prepared following the bleaching method (Porta-de-la-Riva et al., 2012), and put onto RNAi petri plates in triplicate. QPCR was used to identify the efficiency of *RNAi*. The brood size, body length and lifespan of RNAi worms were measured for further analysis as described previously (Wong et al., 1995; Morck and Pilon, 2006).

### Preparation of Hc-DAF-22 Mutant Proteins and Protein Refolding

As previously described, the expression plasmids pET-22b-*Hc-daf-22*-84S (299A/ 349A) were transformed into BL21 (DE3) and induced by 1 mM IPTG at 37°C. Recombinant proteins were purified using Ni-NTA agarose (Qiagen, Shanghai, China) according to manufacturer’s protocol. To refold proteins, the purified eluting solution was added into the lumen space of 30 kDa centrifugal filter (Millipore) and centrifuged at 4°C for 10 min with a speed of 4000 rpm. Then the filtrate was discarded and 0.01 M PBS was added into the filter and centrifugation was repeated under the same condition. This step was repeated for 4–5 times to remove the imidazole. After that, PBS was removed by adding of reaction buffer following the same procedures. The final refolded proteins were kept in reaction buffer and concentrations were measured using the BCA method according to manufacturer’s protocol. Hc-DAf-22 were prepared as control in the subsequent protease activity assay.

### Protease Activity of Hc-DAF-22 Mutant Proteins

To establish a standard curvel, different concentrations of acetoacetyl-CoA (0, 3, 6, 9, 12, 15 μM) were used as substrate to act with 20 mM MgCl_2_, and absorbance at 303 nm were measured. Every 0.1 μg of each protein was added into the thiolase activity determination system (50 mM Tris-Cl (pH 8.1), 20 mM MgCl2, 60 μM CoA and 10 μM AcAc-CoA) and absorbance at 303 nm were measured. The temperature and pH used was 37°C and pH 8.0 which was determined in our previous study.

### Statistical Analyses

Statistical analysis for *Hc-daf-22* mRNA transcription levels and parameters of *C. elegans* were performed using GraghPad Prism 5. *P*-values < 0.05 were considered statistically significant.

## Results

### Preparation of *Hc-daf-22* Mutants

**FIGURE 1:**
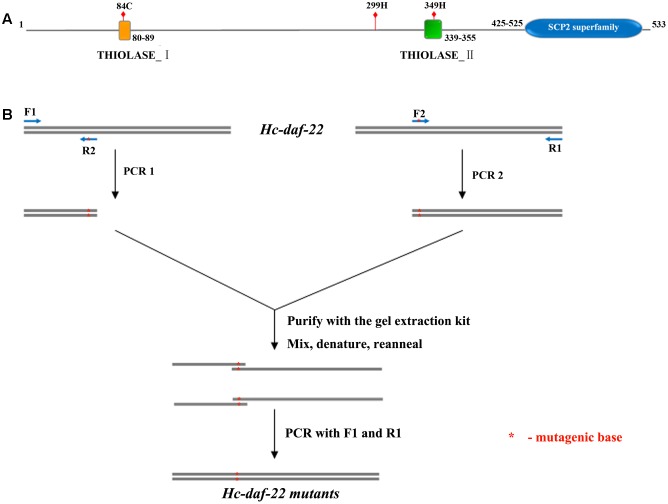
Possible functional sites prediction and site-directed mutagenesis. **(A)** Schematic diagram of Hc-DAF-22 illustrating possible functional sites, conserved domain and motifs; **(B)** detailed representation of introducing site-directed mutagenesis into Hc-daf-22.

**FIGURE 2:**
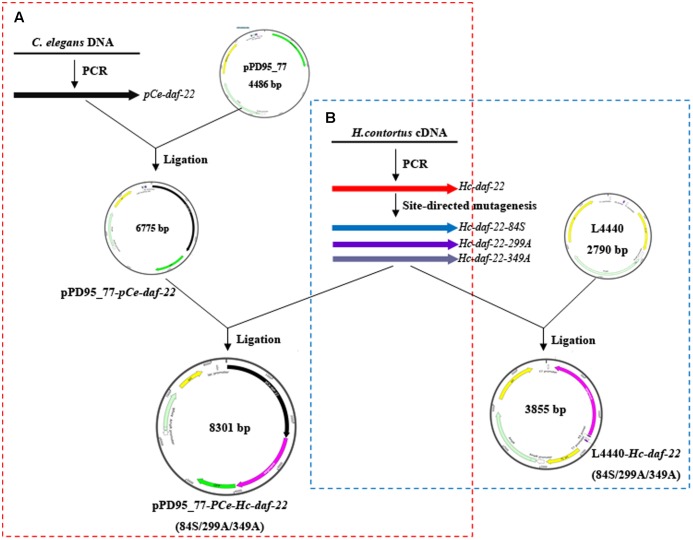
Construction of recombinant plasmids used in rescue and RNAi experiments. **(A)** Cloning *Hc-daf-22* and three mutants into pPD95_77 with *Ce-daf-22* promoter upstream. **(B)** Cloning *Hc-daf-22* and three mutants into L4440.

*Hc-daf-22* full-length coding sequence was cloned from cDNAs produced by reverse transcription of *H. contortus* total RNA and inserted into pET-22b plasmid. This recombinant plasmid pET-22b-*Hc-daf-22* was then used as template in the site-directed mutagenesis experiment and meanwhile as an expression vector to generate Hc-DAF-22. Possible functional sites were predicted by Prosite (**Figure 1**) and 84-cysteine, 299-histidine and 349-histidine were chosen as the target residues as the analysis results showed that 84C and 349H were key active sites of thiolase and 299H was the key site for the modeling of 3-dimension structure. Three mutants of *Hc-daf-22* (pET-22b-*Hc-daf-22*-84S, pET-22b-*Hc-daf-22*-299H and pET-22b-*Hc-daf-22*-349H) were obtained by site-directed mutagenesis using pET-22b-*Hc-daf-22* as a template (**Figure 1**). In the mutants, 84-cysteine was replaced by a serine, 299-histidine and 399-histidine were both replaced by an alanine. For rescue experiments, mutant sequences were amplified from the constructed expression vectors and inserted into pPD95_77 with a *C. elegans daf-22* promoter upstream and a GFP label downstream to construct the microinjection vectors pPD95_77-PCe-*Hc-daf-22* and pPD95_77-PCe-*Hc-daf-22*-84S/299A/349A (**Figure 2A**). For RNAi experiments, mutant sequences were amplified from the constructed expression vectors and inserted into L4440. Thus, L4440-*Hc-daf-22*, L4440-*Hc-daf-22*-84S/299A/349A were obtained (**Figure 2B**).

### *Hc-daf-22* Mutants Failed to Rescue *Ce-daf-22* Mutant *ok693*

**FIGURE 3:**
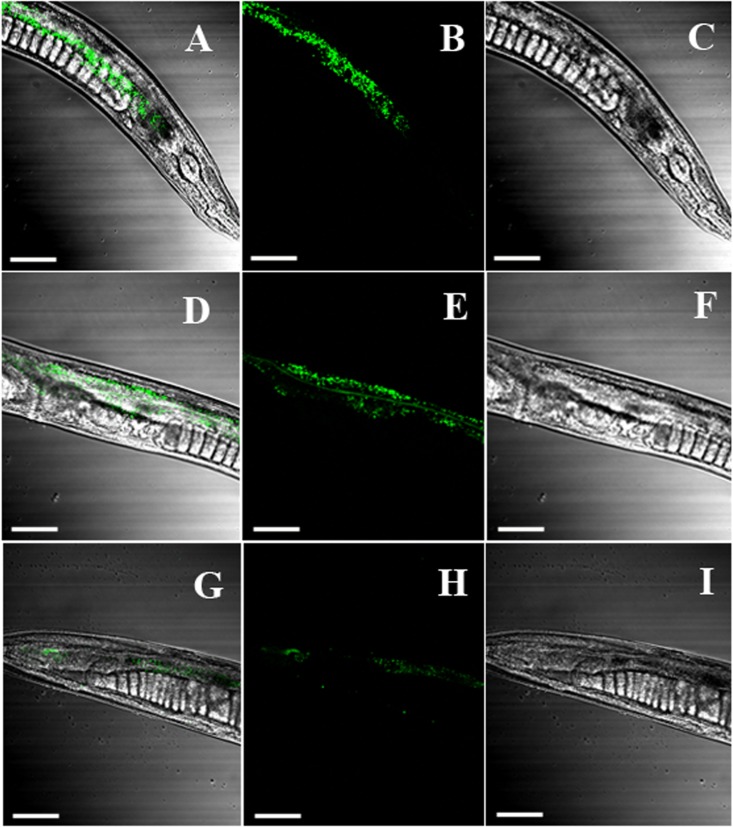
Expression of *Hc-daf-22* mutants in *ok693* strain. *Hc-daf-22* and three mutants were microinjected into the *daf-22* mutant (*ok293*). GFP expression of the F1 or F2 worms was examined under a confocal fluorescence microscopy. The microscopy showed GFP signals were detected in all four transformed lines and the expression patterns were identical. Here only the GFP expression of *Hc-daf-22*-84S were presented as a representation. *Hc-daf-22*-84S was expressed mainly in the intestine and partly in the pharynx and tail. **(A–C)** the view of GFP signal in the pharynx of the transformed worms; **(D–F)** intestine; **(G–I)** tail. *Scale-bars*: 100 μm.

**FIGURE 4:**
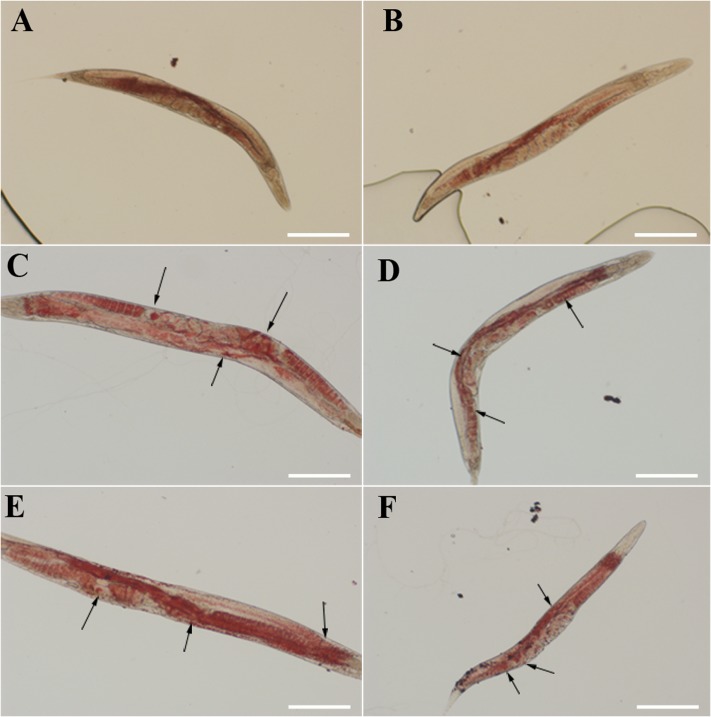
Fat storage in rescued *daf-22* mutant (*ok693*), N2 strain and *ok693* were used as controls. The lipid staining was performed flowing the protocol described in the methods. Worms were placed on a microslide and observed using a differential interference contrast microscope. The figure showed that the fat granules reduced significantly or disappear in the *Hc-daf-22* rescued worms, while there were still many fat granules in the worms of *Hc-daf-22*-84S/299A/349A rescued worms. **(A)** fat storage in N_2_ strain; **(B)** fat storage in *Hc-daf-22* rescued *ok693*; **(C)** fat storage in *Hc-daf-22*-84S rescued *ok693*; **(D)** fat storage in *Hc-daf-22*-299A rescued *ok693*; **(E)** fat storage in *Hc-daf-22*-349 rescued *ok693*; **(F)** fat storage in *daf-22* mutant (*ok693*). Arrows represent fat granules in the worms. *Scale-bars*: 200 μm.

**FIGURE 5:**
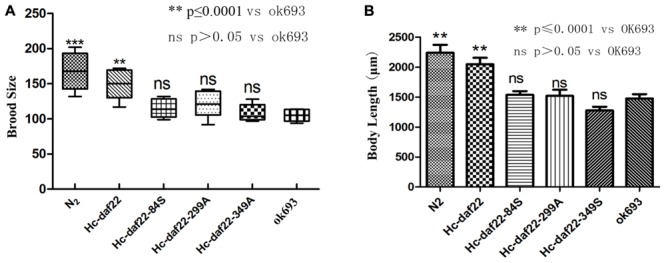
Statistical analysis of brood size and body length of the strains in the rescue experiment, N_2_ strain and *ok693* were used as controls. **(A)** Brood size analysis. Compared to *ok693* strain, *Hc-daf-22* rescued worms showed significant increase in the number of descendant (*P* ≤ 0.0001) but did not reach the level of N_2_ strain, while the other three groups had no significant changes (*P* > 0.05). **(B)** Body length analysis. *Hc-daf-22* transformed worms were significantly longer than *ok693*, while the other three groups had no significant changes which were similar with brood size analysis.

In the rescue experiment, three mutant genes were transformed into *daf-22* mutant strain (*ok693*) while *Hc-daf-22* was used as positive control. To test whether mutations of these possible functional sites could influence the rescue efficiency of *ok693*, lipid staining was conducted, brood size and body length were measured. F1 or F2 *C. elegans* with a left-rolling phenotype were picked out from NGM petri plates and put on a rectangle microslide later covered with a smaller microslide. GFP expression pattern of rolling worms was detected and recorded using a fluorescence microscope. The results showed that all mutant genes and *Hc-daf-22* could be expressed in the transgenic lines under the trigger of *Ce-daf-22* promoter and the expression patterns (position and fluorescence strength) showed no significant difference between *Hc-daf-22* and its three mutants, indicating that mutations of these residues did not influence the transcription and translation of *Hc-daf-22*. The GFP signals of four transgenic lines were detected mainly in the intestine and partly in the pharynx and tail (*Hc-daf-22*-84S expression was shown in **Figure 3**, data of the remaining transformed lines were not shown). Compared to *Hc-daf-22* rescued *ok693* and N_2_ strain, three mutant genes rescued transgenic worms still had huge fat granules in the intestines (**Figure 4**). In addition, *Hc-daf-22* transformed worms were significantly longer than *ok693* but did not reach the length of N_2_ strain, while no significant increase in the body length of the *Hc-daf-22*-84S/299A/349A transformed lines were observed compared to the *Hc-daf-22* transformed worms (**Figure 5**). As the brood size, the increase of capacity in *Hc-daf-22* recued worms were significant compared to *daf-22* mutant strain, while Hc-daf-22-84S/299A/349A rescued lines showed no significant increase in the capacity. In summary, all results above indicated that though mutant *daf-22* genes were overexpressed in the transgenic lines, the biological function of DAF-22 were impaired due to the mutations of the three possible functional sites.

### RNAi Feeding Experiments

**FIGURE 6:**
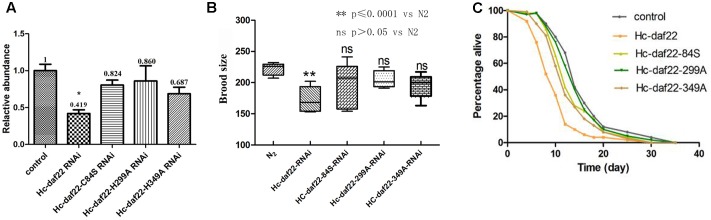
Statistical analysis of brood size and life span of the strains in the RNAi experiment, N2 strain was used as control. RNAi plates were prepared according to standard method and synchronized L1 worms were put on the plates and cultured for certain days. **(A)** Relative quantification of *Ce-daf-22* mRNA in RNAi strains, *Ce-actin-1* was used as reference gene. Compared to N2 strain, *Hc-daf-22* RANi group showed significant decrease of *Ce-daf-22* mRNA while three mutants showing a little decrease but the changes were not significant. **(B)** Brood size analysis of RNAi strains. Egg numbers of *Hc-daf-22* RNAi adults decreased significantly compared to N2 strain, meanwhile mutant groups had no significant changes. **(C)** life span of RNAi strains. Fifty adults in each RNAi group were picked into clear RNAi plates and survival rate were recorded every day until all worms died.

As described above, RNAi experiments were performed to verify whether mutations of possible functional sites could influence the efficiency of *Hc-daf-22* gene in silencing the endogenous *Ce-daf-22* in *C. elegans* N_2_ strain. The relative mRNA levels of *Ce-daf-22* in different RNAi worms were detected by qPCR. As shown in **Figure 6A**, the relative mRNA level was significantly reduced in the N2 strain of the *Hc-daf-22* RNAi group compared to the untreated control group (*t*-test: *t* = 5.796, *df* = 4, *P* = 0.0044), while the relative *Ce-daf-22* mRNA level was not significantly changed after RNAi of *daf-22*-84S (*t*-test: *t* = 1.786, *df* = 4, *P* = 0.1487), similar to *daf-22*-299A RNAi group (*t*-test: *t* = 0.6280, *df* = 4, *P* = 0.5641) and *daf-22*-349A RNAi group (*t*-test: *t* = 2.536, *df* = 4, *P* = 0.0643). The brood size of worms grown on *Hc-daf-22* RNAi plates were significantly reduced compared to N_2_ strain while the three mutant RNAi groups had no significant changes (**Figure 6B**). **Figure 6C** showed there were no significant differences in the life span between the wild strain and the *daf-22* mutant RNAi groups, and the average life span was about 14 days. The survival rate of N_2_ wild type was about 80%, which decreased to 20% or less after feeding on HT115 for 20 days. After feeding on *Hc-daf-22*, the larvae grew slower and the average life span was shorter than that of N_2_ wild type. On the 10th day, the survival rate was less than 40%, and on the 20th day the survival rate of parasites decreased to below 5%. As for the RNAi of *Hc-daf-22* mutants, the survival rates of the worms were all close to 80% after 10 days of RNAi and reduced closely to 20% at 20th days, which was similar to that of N_2_ strain (**Figure 6C**). As for body length analysis, no significant difference was found (data not showed). Therefore, mutations of the above three amino acid sites significantly reduced RNAi efficiency of nature *Hc-daf-22*, indicating that these functional sites were of great importance for Hc-DAF-22 to perform normal biological function.

### Site-Directed Mutagenesis Impaired the Protease Activity of Hc-DAF-22

**FIGURE 7:**
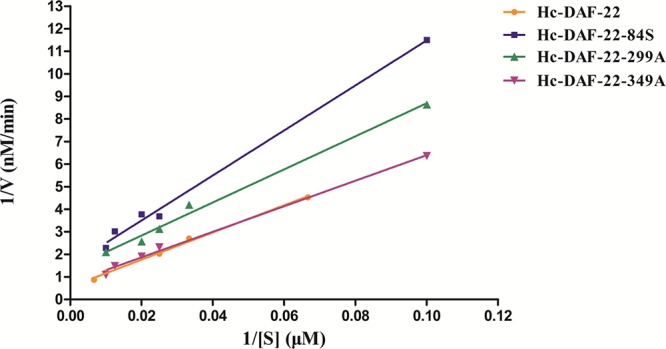
Linewaver–Burke plot of *in vitro* protease activity assay. Linewaver-Burke plot of each protein was drawn according to the reaction rates (V) and corresponding substrate concentrations ([S]). *X* axis represents the value of 1/[S]. The Km value was calculated from the Michaelis equation. Detailed values of Vmax and Km were listed in **Table 2**.

To investigate the changes of thiolase activity between Hc-DAF-22 and three mutants, four proteins were obtained by prokaryotic expression and refolded as described in material and methods. The thiolase activity determination system was used to detect the enzyme activity of each protein. The maximum reaction rate Vmax of each protein was calculated according to the standard curve and Michaelis–Menten constant was obtained from the Linewaver–Burke plot (**Figure 7**). As showed in the figure the Km and Vmax values of Hc-DAF-22 were 33.765 μM and 1784 nM/min respectively (**Table 2**), in contrast, the three mutant proteins all had smaller Vmax and higher Km. We found that 84-cysteine mutation reduced Hc-DAF-22 activity by 62%, 349-histidine mutation reduced the thiolase activity by 58% and 299-histidine mutation reduced the thiolase activity by 33%. The analysis of the kinetic data demonstrated that the mutations of 84/299/349 amino acid had a reduced effect on the activity of thiolase Hc-DAF-22, among which the 84-cysteine mutation exhibited the greatest effect of thiolase activity.

**Table 2:** Vmax and Km values of each protease.

Protein Name	Vmax (nM/min)	Km (μM)
Hc-DAF-22	1784	33.76
Hc-DAF-22-84S	693	51.25
Hc-DAF-22-299A	1387	79.85
Hc-DAF-22-349A	765	78.50

## Discussion

The arrested development diapause is an important strategy of parasite nematodes to survival from extreme conditions (Adams, 1983; Sommerville and Davey, 2002). At present, there are few studies on the metabolic pathways of parasites diapause. In the model organism *C. elegans*, dauer is induced by different kinds of ascarosides with an ascarylose scaffold and fatty-acid-like side chain of different length which are also called the dauer pheromone or daumone synthesized through peroxisomal beta-oxidation pathway catalyzed by four enzyme ACOA-1, MAOC-1, DHS-28 and DAF-22 (Joo et al., 2010; Ludewig and Schroeder, 2013; Butcher, 2017; Ding et al., 2017). Ce-DAF-22 is the key enzyme catalyzing the last step of peroxisomal beta-oxidation pathway. The new gene *Hc-daf-22*, which shares high homology with the *C. elegans* thiolase (*Ce-daf-22*), has been found in *H. contortus*. Conserved domain search had found that Hc-DAF-22 has a functional domain of type I fatty acid thiolase (3-keto-acyl-CoA saloxylase), which is typical in long-chain fatty acid metabolism, indicating that Hc-DAF-22 may have functional resemblance with Ce-DAF-22.

In order to determine the key active amino acid site of Hc-DAF-22 as a thiolase, site-directed mutagenesis was used to introduce single site mutation to the coding sequence of Hc-DAF-22. The results of GFP expression in different transformed lines showed that both *Hc-daf-22* and its three mutants could be transcribed by the *Ce-daf-22* promoter, but there were no differences in the position and GFP fluorescence strength. As seen in **Figure 3**, the GFP signals of four transgenic lines were detected mainly in the intestine and partly in the pharynx and tail in the *ok693* worms. Brood size and body length were measured to confirm the effect of transformed genes on the development of *ok693*. Statistical analysis results showed that the *Hc-daf-22* transgenic worms were significantly longer and laid more eggs than *daf-22* mutant (*ok693*), while *Hc-daf-22*-84S/299A/349A transgenic worms had no significant changes compared to *ok693* (**Figure 5**), which was consistent with the lipid staining result (**Figure 4**). These results illustrated that mutations of the three possible active sites largely reduced the rescue ability of *Hc-daf-22* in *daf-22* mutant (*ok693*). RNAi experiments carried out with *Hc-daf-22* and its mutants in N_2_ strain showed that *Hc-daf-22* could silence the endogenous *Ce-daf-22* while the mutants could not, which was further confirmed by the brood size and life span statistical analysis (**Figure 6**). For the *in vitro* protease activity assay, the analysis of the kinetic data demonstrated that the mutations of 84/299/349 amino acid had a reduced effect on the activity of thiolase Hc-DAF-22, among which the 84-cysteine mutation exhibited the greatest effect of thiolase activity (**Figure 7**).

In our research, we demonstrated that the biological function of *Hc-daf-22* was similar to *Ce-daf-22*, and the 84/299/349 amino acids were key active sites for this thiolase, among which the 84-cysteine was the most important residue. The functional prediction of Hc-DAF-22 showed that 80–89 was a thiolase I motif which was also called 3-ketoacyl-CoA thiolase, and 339–355 was a thiolase II motif (also called Acetoacetyl-CoA thiolase), meanwhile 299H was the key residue that binding these two motifs. 3-ketoacyl-CoA thiolase is mainly involved in degradative pathways such as fatty acid beta-oxidation catalyzing the last step of β-oxidation pathway (Middleton, 1973), whereas Acetoacetyl-CoA thiolase is short-chain-specific biosynthetic thiolase involving in biosynthetic pathways such as poly beta-hydroxybutyrate synthesis or steroid biogenesis (Haapalainen et al., 2007). In *C. elegans*, Ce-DAf-22 participates in the last step of long-chain fatty acids degradation and ascarosides synthesis (Gallegos et al., 2001). The rescue and RNAi experiments results showed that Hc-DAF-22 was conserved in protein function with Ce-DAF-22, suggesting that thiolase I rather than thiolase II was the predominate functional motif in Hc-DAF-22. This would explain why mutation in 84C impaired the protease activity more severely than 349H.

Although *C. elegans* was a widely used model organism in parasites research due to its close evolutionary relationship and conserved gene functions with parasitic nematodes, it might also be noted that the life cycles and survival environments of free living nematodes and parasitic nematodes were significantly different, indicating that *C. elegans* would be a useful but not a sufficient tool in functional analysis of parasites and further work should be done on the parasites to confirm the results derived from model organism during which other different results would probably arise. In this study, the function of Hc-DAF-22 was mainly investigated via *C. elegans* and *in vitro* protease activity assay. In order to validate whether the results were identical in *H. contortus*, rescue and RNAi experiments should be carried out using *H. contortus*. Overall, more knowledge of metabolic mechanism should be further investigated if diapause is chosen to be a new target for the control and prevention of haemonchosis.

## Author Contributiions

YH and XZ finished the site-directed mutagenesis of *Hc-daf-22*, construction of all recombinant plasmids, completed the functional rescue test of *daf-22* mutant (*ok693*) with *Hc-daf-22* mutants, performed the RNAi, and YH drafted this manuscript, these two authors had equal contribution to this research. HZ finished the isolation of *Hc-daf-22*, performed the relative quantification PCR, brood size and life span record of RNAi. HD obtained four proteins and conducted protein refolding and the *in vitro* protease activity assy. XG was responsible for recording the brood size and body length in the rescue experiment. YY did the fat staining and GFP expression examination. QZ was responsible for possible functional active site prediction. XC performed the statistical analysis of all data, AD was the corresponding author. All authors read and approved the submitted version.

## Conflict of Interest Statement

The authors declare that the research was conducted in the absence of any commercial or financial relationships that could be construed as a potential conflict of interest.

## Footnotes

1 http://prosite.expasy.org/prosite.html
